# Ambulatory Blood Pressure Comparison of Amlodipine, Cilnidipine, and Azelnidipine in Hypertension: The AMCILAZ-EH Multicentre, Prospective, Observational Study

**DOI:** 10.7759/cureus.110009

**Published:** 2026-05-31

**Authors:** Prabhakar Dorairaj, Murugarajan Singaram, Narasingan SN, Harikrishnan Parthasarathy, Natarajan Srikant, Shanmugasundaram Somasundaram

**Affiliations:** 1 Cardiology, Ashwin Clinic, Chennai, IND; 2 Cardiology, Sri Kannan Heart Care Centre, Chennai, IND; 3 Internal Medicine, SNN Clinic, Chennai, IND; 4 Cardiology, Shri Krishnasai Clinic, Chennai, IND; 5 Oral Pathology and Microbiology, Manipal College of Dental Sciences, Manipal University, Mangalore, IND; 6 Cardiology, Billroth Hospitals, Chennai, IND

**Keywords:** ambulatory blood pressure monitoring, amlodipine, azelnidipine, calcium channel blockers, cilnidipine, circadian blood pressure, hypertension

## Abstract

Background

Calcium channel blockers (CCBs) are recommended as first-line therapy for hypertension (HTN). Amlodipine is well established, while newer agents such as cilnidipine and azelnidipine possess additional pharmacologic properties that may influence ambulatory blood pressure (BP) and circadian patterns. The clinical relevance of these differences in real-world practice remains uncertain.

Methods

A multicentric, prospective, observational study included adults with mild-to-moderate essential HTN, in which patients received amlodipine (5-10 mg), cilnidipine (10-20 mg), or azelnidipine (8-16 mg). Ambulatory blood pressure monitoring (ABPM) and clinic BP were recorded at baseline and after eight weeks.

Primary endpoints were changes in 24-hour, awake, and asleep BP (systolic blood pressure (SBP)/diastolic blood pressure (DBP)) by ABPM and clinic BP. Secondary endpoints included dipping status, target BP achievement, and pulse rate changes. Continuous variables were analysed using paired t-tests and one-way ANOVA; categorical variables were analysed using Fisher’s exact test.

Results

After screening 113 patients, 50 patients were included in the final analysis (amlodipine n = 17, cilnidipine n = 12, azelnidipine n = 21). All groups demonstrated significant reductions in clinic and ambulatory BP (p < 0.001). Mean 24-hour SBP reductions were -20.2 ± 12.7 mmHg, -14 ± 10.2 mmHg, and -12.8 ± 9.6 mmHg, respectively, with no significant intergroup difference (p = 0.111). However, awake ambulatory SBP (p = 0.047) and DBP (p = 0.034) showed significant intergroup differences, primarily between amlodipine and azelnidipine. All other parameters, including 24-hour and asleep BP, pulse rate, and dipping status, were comparable.

Target BP achievement and pulse rate changes were comparable. No significant change in dipping pattern was observed, although azelnidipine showed a numerical improvement. Baseline characteristics between completers and excluded patients were comparable, suggesting minimal attrition bias.

Conclusions

All three CCBs demonstrated a significant reduction in BP, with comparable short-term efficacy. No consistent intergroup differences were observed across most parameters, except for isolated differences in awake ambulatory BP. However, these findings should be interpreted in the context of the limited sample size and reduced statistical power. Larger randomized studies with possible home BP monitoring are required.

## Introduction

Hypertension (HTN) remains a leading contributor to global cardiovascular morbidity and mortality. Global HTN guidelines recommend calcium channel blockers (CCBs) as one of the first-line medications because of their proven efficacy and favourable tolerability profile [1,2]. Amlodipine is the most extensively studied long-acting dihydropyridine CCB with established cardiovascular outcome benefits [3]. Amlodipine works by inhibiting the transmembrane influx of calcium by blocking the L-type calcium channels in the vascular smooth muscle and heart. Amlodipine has high vascular selectivity and acts predominantly on vascular smooth muscle [4]. Newer agents such as cilnidipine and azelnidipine have been developed with additional pharmacodynamic properties. Cilnidipine inhibits both L-type and N-type calcium channels and may attenuate sympathetic activity, while azelnidipine blocks the L-type and T-type calcium channels. The T-type channel blockade is associated with an additional reduction in aldosterone secretion. The newer CCBs have been shown to reduce blood pressure (BP) and heart rate. Additional improvement in urinary albumin excretion has been shown in some studies. CCBs are generally considered safe antihypertensive agents. They can be used safely in a wide spectrum of patients, such as those with obstructive airway disease, peripheral vascular disease, diabetes mellitus, and kidney disease. Their side-effect profile is comparable to placebo apart from leg swelling, which is variably reported among CCBs. Leg swelling is reversible, and newer CCBs have a lower propensity to cause it [5,6].

Ambulatory blood pressure monitoring (ABPM) provides superior prognostic information compared with clinic BP, particularly for nocturnal HTN and circadian BP variation [7]. Whether pharmacologic differences among CCBs translate into clinically meaningful ambulatory BP advantages remains uncertain. The BP-lowering effects of CCBs have not been compared systematically. Since guidelines emphasize achieving target BP as an important step in the management of HTN, a comparison of CCBs may provide practical guidance on their comparative efficacy. The present study hypothesized that the newer CCBs - cilnidipine and azelnidipine - were superior to amlodipine in office and 24-hour BP control, with better dipping patterns. Several parameters used in routine clinical practice - office BP (OBP), ABPM parameters (awake, 24-hour, asleep BP), dipping pattern on ABPM, pulse rate (in OBP and ABPM), and achievement of target BP - were studied after eight weeks of therapy. The study is a non-randomized, real-world exploratory study in which the physician exercised discretion regarding medication dosage.

## Materials and methods

Study design

A comparative, observational, parallel-arm, longitudinal, multicentric, open-label study was conducted in adult patients aged 18-80 years with essential HTN treated in routine outpatient practice. Stable patients with an OBP of ≥140/90 mmHg, either untreated or on the same medication for four weeks, were included. Patients with additional cardiovascular risk factors, including type 2 diabetes mellitus, left ventricular dysfunction, or chronic kidney disease (< stage 3), were eligible if clinically stable. Essentially, patients should not be on beta-blockers or dihydropyridine CCBs. Both treatment-naïve and known hypertensives were included. The following patients were excluded: uncontrolled BP ≥180/110 mmHg, irregular heart rhythm such as atrial fibrillation, CKD stage 3 or higher, secondary HTN (including renal artery stenosis and pheochromocytoma), type 1 diabetes mellitus, active liver disease, thyroid disease, ≥ moderate valvular heart disease, pericardial disease, unstable heart disease, heart failure, and pregnant women or those planning pregnancy.

Treatment groups and study protocol

Patients received physician-directed therapy with amlodipine 5-10 mg daily, cilnidipine 10-20 mg daily, or azelnidipine 8-16 mg daily. Treatment allocation was based on physician discretion in routine clinical practice and was not randomized. Physicians were advised to initiate consecutive patients on the three drugs consecutively. Dose selection and escalation were individualized based on BP response and tolerability, without a predefined titration protocol. This was based on the clinical judgment of the physician and the BP response to the initial dose.

During the initial office screening, demographic details, comorbidities, and details of other medications were collected. Clinic BP was measured using STRIDE-approved standardized automated A&D UA-651 BP apparatus after five minutes of seated rest, followed by two further readings a minute apart. Patients underwent ABPM at baseline using a STRIDE-approved ABPM apparatus (A&D TM-2440). Open-label CCB therapy was initiated.

At an optional first follow-up at four weeks, the physician could escalate the dose. Medication adherence was assessed by patient self-report during follow-up visits. After eight weeks, compliance was ensured by patient enquiry, and the patient underwent a second ABPM. During treatment, adverse effects, if any, were also recorded. Patients who did not complete the second ABPM, were noncompliant, or were lost to follow-up were excluded.

The primary endpoints were: (i) change in 24-hour, awake, and asleep BP (systolic blood pressure (SBP)/diastolic blood pressure (DBP)) between baseline and eight weeks for the three arms by ABPM; (ii) change in OBP (SBP/DBP) between baseline and eighth week for all three arms. Secondary endpoints were: (i) change in dipping status by ABPM in each arm over eight weeks; (ii) proportion of patients achieving target awake BP of <135/85 mmHg using ABPM; (iii) proportion of patients with treatment-emergent adverse events during the observation period in all three arms; (iv) change in pulse rate in office and ABPM between the three arms. Missing data were not imputed; analyses were performed on available data, and patients with incomplete follow-up or ABPM were excluded.

The study was approved by the Institutional Ethics Committee (ECR/1761/Inst/TN/2023) and registered in the Clinical Trials Registry of India (CTRI/2024/08/072999). Informed consent was obtained from each patient.

Statistical analysis

Data were summarized using descriptive statistics. Continuous variables were expressed as mean ± standard deviation. The normality of data distribution was assessed using the Shapiro-Wilk test. A p-value greater than 0.05 was considered indicative of normal distribution. Intergroup comparisons were performed using one-way analysis of variance (ANOVA), and intragroup comparisons were carried out using paired t-tests. Categorical variables were analysed using Fisher’s exact test. A two-tailed p-value <0.05 was considered statistically significant.

Although a small proportion of variables (~25%) showed deviation from normality (p < 0.05), these deviations were sporadic. Given the relatively small sample sizes and the robustness of parametric tests to mild violations of normality, particularly when group sizes are comparable, parametric methods were still considered appropriate. Furthermore, key outcome variables and primary endpoints largely satisfied normality assumptions, reinforcing the validity of parametric analysis. For variables with evident skewness, results were interpreted with caution, and where required, non-parametric equivalents were considered to confirm consistency of findings.

The sample size was determined based on the requirement to detect a clinically significant intergroup difference of 5 mmHg in SBP, assuming a standard deviation of 10 mmHg. Sample size estimation was performed using an analysis of covariance (ANCOVA) framework to account for baseline-to-endpoint correlation. Assuming a clinically meaningful difference of 5 mmHg in SBP between groups, a standard deviation of 10 mmHg, a two-sided alpha level of 0.05, and 80% power, a minimum of 108 evaluable patients (36 per group) was required. To account for potential attrition, including loss to follow-up and non-compliance, the recruitment target was increased by approximately 28%, resulting in a planned sample size of 150 patients (50 per group). Due to real-world constraints, the final analysed sample was smaller, and the study should therefore be considered exploratory.

## Results

**Figure 1:**
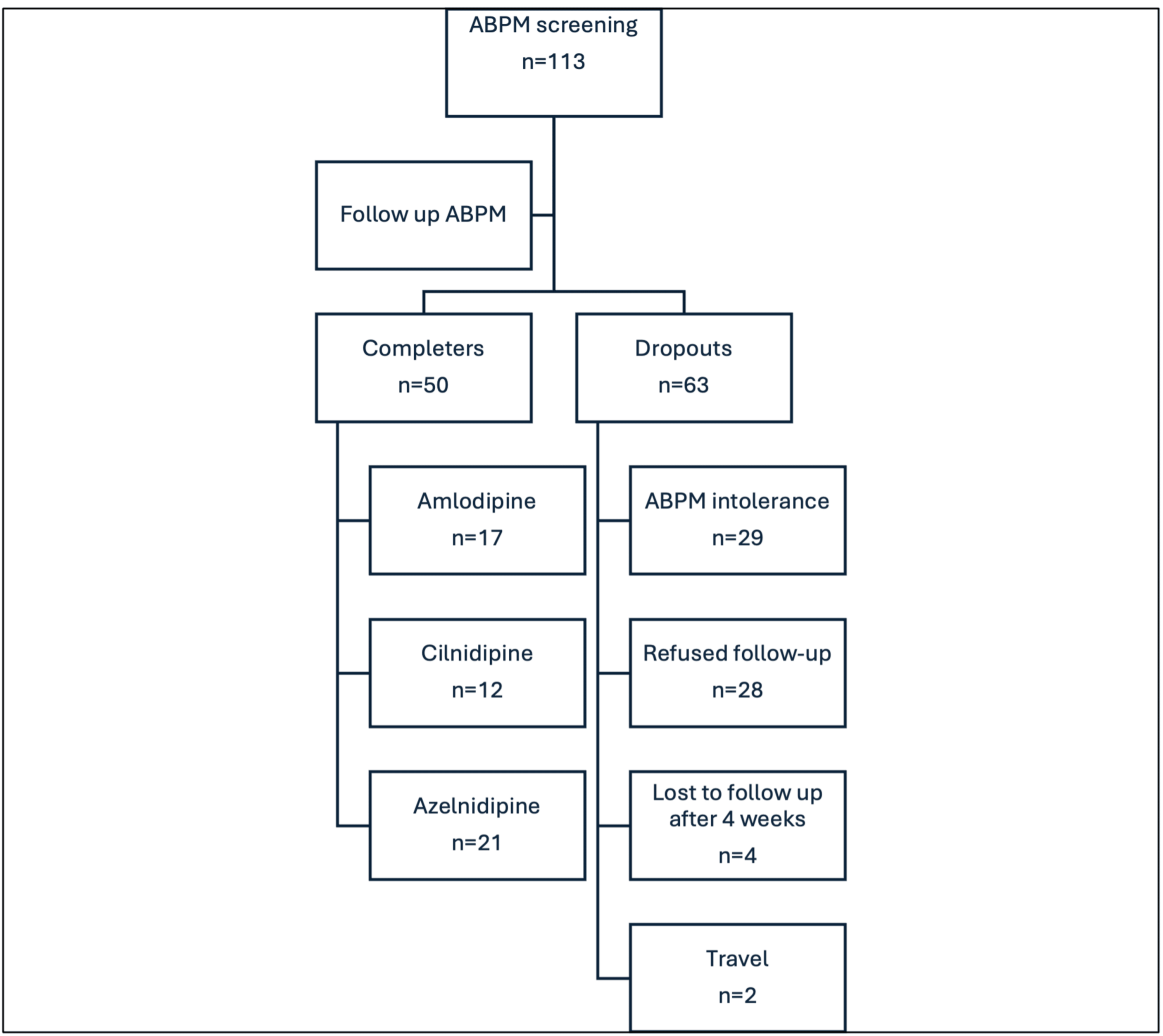
Study flow diagram of patient screening, follow-up, and final analysis in the AMCILAZ-EH study Patients undergoing ABPM screening were followed prospectively over eight weeks. Of 113 screened patients, 50 completed follow-up and were included in the final analysis (amlodipine n = 17, cilnidipine n = 12, azelnidipine n = 21), while 63 patients were excluded or discontinued follow-up. Reasons for exclusion included ABPM intolerance (n = 29), refusal of follow-up ABPM (n = 28), loss to follow-up after four weeks (n = 4), and travel-related discontinuation (n = 2). ABPM: ambulatory blood pressure monitoring; AMCILAZ-EH: AMlodipine CILnidipine AZelnidipine in Essential Hypertension

ABPM screening was performed in 113 patients from six centres. Following loss to follow-up and non-compliance, only 50 patients (44.2%) completed the study. The study was terminated early due to high attrition, and analysis was performed on the available cohort. The final analysis included 50 patients in the AMCILAZ-EH (AMlodipine CILnidipine AZelnidipine in Essential Hypertension) study. In the final analysis, amlodipine, cilnidipine, and azelnidipine were prescribed in 17, 12, and 21 patients, respectively. The study flow is shown in Figure 1.

Baseline data between the three groups were comparable, and there was no statistically significant difference between the groups. The baseline data are summarized in Table 1.

**Table 1:** Baseline data of the three groups of patients across the treatment groups Baseline characteristics of patients across treatment groups. Continuous variables are expressed as mean ± standard deviation and compared using one-way analysis of variance (ANOVA), with F-values reported. Categorical variables are expressed as percentages and compared using the chi-square (χ²) test or Fisher’s exact test, where appropriate. SBP: systolic blood pressure; DBP: diastolic blood pressure

Variable	Amlodipine (n=17)	Cilnidipine (n=12)	Azelnidipine (n=21)	Test statistic	p-value
Age (years)	51.4 ± 13.9	47.8 ± 13.5	51.5 ± 13.4	F = 0.39	0.68
Male sex (%)	52.9%	58.3%	52.4%	χ² = 0.13	0.94
Clinic SBP (mmHg)	153.9 ± 15.0	158.2 ± 13.6	155.6 ± 16.0	F = 0.30	0.74
Clinic DBP (mmHg)	94.6 ± 10.8	96.2 ± 9.8	96.9 ± 9.3	F = 0.21	0.81
Pulse rate (bpm)	84.8 ± 10.7	78.8 ± 10.8	80.1 ± 9.8	F = 1.17	0.32
Diabetes mellitus (%)	29.4%	16.7%	19.0%	χ² = 0.92	0.63
Dyslipidaemia (%)	58.8%	58.3%	38.1%	χ² = 1.51	0.47
Smoking (%)	5.9%	8.3%	9.5%	χ² = 0.16	0.92
Coronary artery disease (%)	5.9%	8.3%	9.5%	χ² = 0.19	0.91
Chronic kidney disease < stage 3 (%)	0%	0%	0%	-	-

At the completion of the eight-week ambulatory BP assessment, the mean achieved doses were 6.3 ± 2.2 mg for amlodipine, 13.6 ± 5.0 mg for cilnidipine, and 13.6 ± 3.8 mg for azelnidipine. Although sample size estimation was based on an ANCOVA framework, the final analysis was limited to unadjusted comparisons due to the modest sample size. Multivariable or covariate-adjusted analyses were not performed, and therefore, residual confounding due to baseline differences, treatment allocation, and comorbidities cannot be excluded. The primary endpoints of all three arms showed significant intragroup variation at eight weeks but no intergroup statistical difference by one-way ANOVA, as shown in Table 2. All within-group reductions were statistically significant (p < 0.001).

**Table 2:** Primary endpoints - change in ambulatory and office blood pressure among the treatment groups at eight weeks Changes in ambulatory and clinic blood pressure parameters at eight weeks. Values are expressed as mean ± standard deviation. Intergroup comparisons were performed using one-way analysis of variance (ANOVA), with F-values reported. *p < 0.05 considered statistically significant. SBP: systolic blood pressure; DBP: diastolic blood pressure; ABPM: ambulatory blood pressure monitoring

Parameter	Amlodipine (n = 17)	Cilnidipine (n = 12)	Azelnidipine (n = 21)	F value	p-value*
24-hour SBP (mmHg)	-20.2 ± 12.7	-14 ± 10.2	-12.8 ± 9.6	2.3064	0.111
24-hour DBP (mmHg)	-10.8 ± 5.6	-6.9 ± 5.4	-7.19 ± 6.2	2.2807	0.113
Awake SBP (mmHg)	-21.1 ± 12.3	-14.417 ± 9.5	-12.1 ± 10.4	3.2663	0.047
Awake DBP (mmHg)	-11.5 ± 6.7	-6.75 ± 5.8	-6.4 ± 6.0	3.6432	0.034
Asleep SBP (mmHg)	-17.6 ± 17.2	-17 ± 16.3	-13.8 ± 12.4	0.3457	0.71
Asleep DBP (mmHg)	-9.2 ± 8.6	-8.4 ± 8.0	-7.4 ± 9.0	0.1911	0.827
Office SBP (mmHg)	-22 ± 14.1	-17.5 ± 16.4	-17.2 ± 13.6	0.5462	0.583
Office DBP (mmHg)	-9.5 ± 6.7	-8.1 ± 7.9	-10.4 ± 7.9	0.3052	0.739

Following the intergroup comparison, one-way ANOVA with Tukey post-hoc analysis was performed, and the results are given in Table 3.

**Table 3:** Tukey post-hoc analysis of intergroup differences in ambulatory and office blood pressure parameters Values represent mean differences between treatment groups derived from Tukey post-hoc analysis following one-way analysis of variance (ANOVA). Positive values indicate a greater reduction in the first-mentioned treatment group. Statistically significant intergroup differences were observed only for awake SBP and awake DBP between amlodipine and azelnidipine. *p < 0.05 considered statistically significant. SBP: systolic blood pressure; DBP: diastolic blood pressure

Parameter	Amlodipine vs Cilnidipine		Amlodipine vs Azelnidipine		Azelnidipine vs Cilnidipine	
	Mean difference	p-value*	Mean difference	p-value*	Mean difference	p-value*
24-hour SBP	6.24	0.296	7.38	0.109	-1.14	0.955
24-hour DBP	3.907	0.192	3.63	0.150	0.274	0.991
Awake SBP	6.70	0.247	8.97	0.040	-2.27	0.835
Awake DBP	4.838	0.112	5.16	0.039	-0.321	0.989
Asleep SBP	0.688	0.992	3.88	0.720	-3.190	0.829
Asleep DBP	0.833	0.966	1.77	0.812	-0.940	0.952
Office SBP	4.455	0.713	4.71	0.592	-0.260	0.999
Office DBP	1.35	0.895	-0.867	0.940	2.22	0.717

For clinic BP parameters, there was no statistically significant difference between the three groups at baseline for SBP (p = 0.775) or DBP (p = 0.793), indicating comparability of groups prior to intervention. Similarly, post-treatment SBP (p = 0.284) and DBP (p = 0.486), as well as the change in SBP (p = 0.583) and DBP (p = 0.739), did not show statistically significant intergroup differences, suggesting comparable antihypertensive effects across all three drugs (0% statistically significant findings in clinic BP parameters). For clinic pulse rate, no significant differences were observed between the groups at baseline (p = 0.281), post-treatment (p = 0.533), or in the change values (p = 0.332), indicating uniform effects across groups (0% significance).

For 24-hour ambulatory BP parameters, both SBP and DBP showed no statistically significant differences across groups at baseline, post-treatment, or in change values (all p > 0.05), indicating similar efficacy in controlling ambulatory BP (0% significant differences). For awake ambulatory SBP, although baseline (p = 0.564) and post-treatment values (p = 0.312) were comparable, the change in awake SBP showed a statistically significant difference between groups (p = 0.047). Post-hoc analysis revealed that this difference was primarily between amlodipine and azelnidipine (mean difference = 8.97, p = 0.040), while other pairwise comparisons were not significant. Thus, only one parameter (~2-3% of total comparisons) demonstrated a statistically significant intergroup difference. For awake DBP, the difference across groups was also statistically significant (p = 0.034). Tukey post-hoc analysis showed a significant difference between amlodipine and azelnidipine (mean difference = 5.16, p = 0.039), with no significant differences involving cilnidipine. This represents another isolated significant finding. For other ambulatory parameters, including pulse rate, night-time BP, dipping patterns, and dipping percentages, no statistically significant differences were observed between groups (all p > 0.05), indicating consistent effects across all treatment arms.

Overall interpretation

Overall, intergroup comparison revealed that the three antihypertensive agents demonstrated good efficacy across the majority of clinical and ambulatory BP parameters, with most comparisons not demonstrating statistically significant intergroup differences (p > 0.05). Only a small proportion of parameters (~5%) - specifically the change in awake SBP and DBP - showed statistically significant differences, primarily between amlodipine and azelnidipine. The absence of statistically significant differences should not be interpreted as evidence of equivalence, particularly given the limited statistical power of the study.

Secondary End-Point Analysis

The proportion of patients achieving clinic BP ≤140/90 mmHg was numerically higher in the cilnidipine group (83.3%) compared to amlodipine (52.9%) and azelnidipine (42.9%), but this did not achieve statistical significance. Secondary endpoints across the different treatment groups are given in Table 4.

**Table 4:** Secondary end points across the treatment groups Secondary endpoints, including target blood pressure achievement and pulse rate changes. Categorical variables were compared using Fisher’s exact test (χ² values shown), and continuous variables using one-way analysis of variance (ANOVA) (F-values shown). Values are expressed as mean ± standard deviation or number (%).

Parameter	Amlodipine (n = 17)	Cilnidipine (n = 12)	Azelnidipine (n = 21)	χ² value	p-value
Clinic BP ≤140/90 (%)	9 (52.9%)	10 (83.3%)	9 (42.9%)	2.48	0.29
Awake ABPM ≤135/85 (%)	11 (64.7%)	7 (58.3%)	9 (42.9%)	2.05	0.36
Change in pulse rate (office)	-4.2 ± 11.7	+1.4 ± 7.8	-3.6 ± 9.6	1.35 (F)	0.27
Change in pulse rate (ABPM)	-3.3 ± 7.6	-2.2 ± 5.9	-5.2 ± 6.9	0.85 (F)	0.44

**Figure 2:**
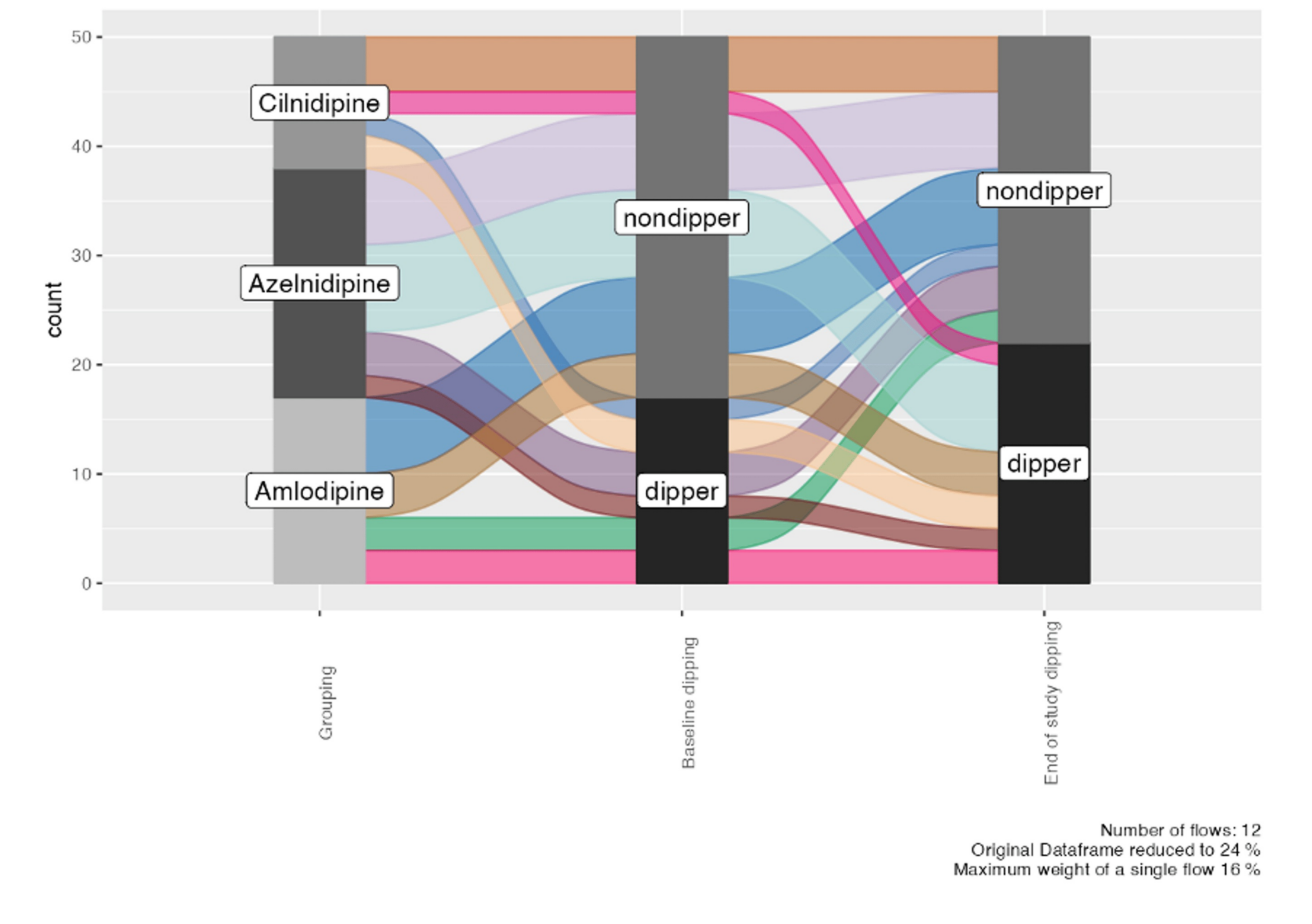
Alluvial plot showing transition in dipping status from baseline to eight weeks across treatment groups This alluvial plot illustrates the distribution and transition of patients across dipping categories (dipper and non-dipper) from baseline to the end of the eight-week study period, stratified by treatment group (amlodipine, cilnidipine, and azelnidipine). Each vertical bar represents the total number of patients in each category at a given stage (grouping, baseline dipping status, and end-of-study dipping status). The connecting flows represent patient-level transitions between categories, with the width of each flow proportional to the number of patients undergoing that transition. Dipper status was defined as a ≥10% reduction in asleep systolic blood pressure compared with awake systolic blood pressure on ABPM. The plot demonstrates that most patients remained in their baseline category, while a greater proportion of transitions from non-dipper to dipper status were observed in the azelnidipine group. ABPM: ambulatory blood pressure monitoring

The baseline dippers and end-of-study dippers are given in Table 5. Within-group analysis using McNemar’s test demonstrated no statistically significant change in dipping status within any treatment group, although a numerical increase in dippers was observed in the azelnidipine group (p = 0.13). The alluvial plot of the dipper and non-dipper is shown in Figure 2.

**Table 5:** Comparison of the baseline dipping status and the end of study dipping between treatment groups Changes in dipping status from baseline to eight weeks. Improved indicates conversion from non-dipper to dipper. Worsened indicates dipper to non-dipper. Within-group comparisons were performed using McNemar’s test for paired categorical data, which did not show any statistical change within the groups. *p < 0.05 considered statistically significant.

Drug	n	Baseline dippers, n (%)	End-of-study dippers, n (%)	Improved (n)	Worsened (n)	McNemar χ²	McNemar p-value*
Amlodipine	17	6 (35.3%)	7 (41.2%)	1	0	0.00	1.00
Cilnidipine	12	5 (41.7%)	5 (41.7%)	0	0	-	1.00
Azelnidipine	21	6 (28.6%)	10 (47.6%)	4	0	2.25	0.13

Analysis of why the study did not reach the requisite numbers

Originally, the study was scheduled to be conducted in eight centres following Ethics committee approval, out of which two centres did not enrol any patients. Among the 63 excluded patients, 29 declined repeat ABPM due to discomfort, 28 refused follow-up, four did not return after the four-week visit, and two were unavailable due to travel. This highlights the limited tolerability of repeated ABPM in real-world practice. A sensitivity analysis was done on baseline characteristics, which were comparable between completers and excluded patients, and it demonstrated no significant differences in age, clinic SBP, clinic DBP, or most baseline ABPM parameters. However, excluded patients had modestly lower baseline 24-hour SBP (137.9 ± 15.2 vs 144.1 ± 14.3 mmHg; p = 0.030) and awake SBP (140.7 ± 16.1 vs 147.1 ± 13.8 mmHg; p = 0.028) compared with completers (Table 6).

**Table 6:** Sensitivity analysis between completers and excluded patients Sensitivity analysis comparing baseline characteristics between completers and excluded patients. Values are expressed as mean ± standard deviation. Comparisons were performed using independent samples t-test with Welch’s correction. t-values and corresponding p-values are reported. SBP: systolic blood pressure; DBP: diastolic blood pressure

Variable	Completers (n = 50)	Dropouts (n = 63)	t-value	p-value
Age (years)	50.6 ± 13.4	50.8 ± 13.2	-0.08	0.99
Clinic SBP (mmHg)	155.6 ± 14.9	153.9 ± 14.7	0.61	0.27
Clinic DBP (mmHg)	96.0 ± 9.7	94.3 ± 9.6	0.93	0.37
Pulse rate (bpm)	81.3 ± 10.4	86.4 ± 12.2	-1.62	0.11
24-hour SBP (mmHg)	144.1 ± 14.3	137.9 ± 15.2	2.21	0.030
24-hour DBP (mmHg)	88.9 ± 8.2	86.9 ± 10.9	1.11	0.266
Awake SBP (mmHg)	147.1 ± 13.8	140.7 ± 16.1	2.23	0.028
Awake DBP (mmHg)	91.3 ± 8.8	88.8 ± 11.3	1.29	0.198
Asleep SBP (mmHg)	137.7 ± 19.3	133.4 ± 16.5	1.26	0.212
Asleep DBP (mmHg)	83.5 ± 9.8	83.8 ± 10.7	-0.13	0.893

## Discussion

Most of the guidelines agree that the control of BP is the most important part of the management of HTN [8]. The AMCILAZ-EH study demonstrated significant reductions in both clinic and ambulatory BP across all three treatment groups over eight weeks. Although isolated differences were observed in awake ambulatory BP, no consistent intergroup differences were seen across most BP parameters. Overall, the findings suggest that amlodipine, cilnidipine, and azelnidipine provide broadly similar short-term BP reduction in routine clinical practice. However, these observations should be interpreted cautiously, given the observational design and modest sample size.

A major strength of the study was the use of ABPM, which enabled assessment of 24-hour, awake, and asleep BP patterns beyond conventional office measurements. While awake, ambulatory SBP and DBP showed statistically significant differences between some treatment groups, these findings were modest and not consistently reflected in 24-hour or asleep BP measurements. The clinical significance of these isolated findings remains uncertain. The observed variation may reflect differences in pharmacodynamic profile, diurnal drug action, or simply variability related to the small sample size. Importantly, the absence of statistically significant differences across most parameters should not be interpreted as evidence of equivalence.

The magnitude of SBP reduction observed in this study (13-19 mmHg by ABPM) was clinically meaningful and comparable to reductions reported in earlier studies [9] evaluating dihydropyridine CCBs. Two crossover studies comparing cilnidipine and azelnidipine similarly reported no major differences in BP reduction [10,11]. A numerically greater reduction in SBP was observed with amlodipine, although this did not reach statistical significance. Previous randomized and observational studies have also generally reported no major differences in BP reduction between amlodipine, cilnidipine, and azelnidipine in routine clinical settings (Table 7). Although cilnidipine and azelnidipine possess additional pharmacologic properties, these did not translate into clear advantages in circadian BP control, restoration of dipping pattern, or pulse rate reduction in the present study. A multicentre retrospective study studying renal outcomes noted better SBP and DBP reduction with amlodipine compared to other CCBs [12]; however, such comparisons should be interpreted cautiously given differences in study design and populations.

**Table 7:** Comparative studies of calcium channel blockers Summarizing prior randomized and observational studies evaluating BP reduction among dihydropyridine calcium channel blockers, there was no clinically meaningful difference in blood pressure reduction among dihydropyridine calcium channel blockers in real-world settings - this should be interpreted with caution, considering the small final sample size. BP: blood pressure; ABPM: ambulatory blood pressure monitoring; SBP: systolic blood pressure; DBP: diastolic blood pressure; T2DM: type 2 diabetes mellitus; EMR: electronic medical record; DHP-CCB: dihydropyridine calcium channel blocker; AMCILAZ-EH: AMlodipine CILnidipine AZelnidipine in Essential Hypertension

Study	Design/population	Comparison	ΔSBP (mmHg), mean ± SD	ΔDBP (mmHg), mean ± SD	Mean difference (SBP)	95% CI	p-value	Key additional findings
Jadhav et al. (2021) [13]	Real-world EMR	Amlodipine vs other DHP-CCBs	Comparable	Comparable	~0	NS	NS	No BP superiority
Hoshide et al. (2005) [14]	RCT, ABPM	Cilnidipine vs Amlodipine	-15.2 ± 6.8 vs -14.6 ± 7.1	-10.8 ± 5.4 vs -10.2 ± 5.6	-0.6	(-2.5 to 1.3)	NS	↓ HR with cilnidipine
Adake et al. (2015) [15]	Prospective	Cilnidipine vs Amlodipine	-21.1 ± 9.2 vs -23.1 ± 8.8	-10.3 vs -12.5 ± 9.6	-2.0	NS	NS	↓ Oedema with cilnidipine
Dharapur and Patil (2019) [16]	Comparative	Cilnidipine vs Amlodipine	-20.3 ± 9.1 vs -17.8 ± 8.7	-12.0 ± 6.8 vs -11.5 ± 6.5	-2.5	(-5.1 to 0.1)	0.06	Trend toward better SBP reduction
Abe et al. (2013) [10]	RCT (T2DM HTN)	Cilnidipine vs Azelnidipine	-16.4 ± 7.3 vs -15.9 ± 7.0	-9.8 ± 5.5 vs -9.5 ± 5.2	-0.5	(-2.8 to 1.8)	NS	↓ Albuminuria with cilnidipine
Kuramoto et al. (2003) [17]	ABPM	Azelnidipine vs Amlodipine	-14.7 ± 6.9 vs -15.1 ± 7.2	-9.6 ± 5.2 vs -9.9 ± 5.4	0.4	(-1.9 to 2.7)	NS	Smoother BP with azelnidipine
Khan (2023) [11]	Prospective (T2DM HTN)	Cilnidipine vs Azelnidipine	-17.8 ± ~7 vs -16.9 ± ~7	-10.5 ± ~5 vs -10.1 ± ~5	-0.9	(approx NS)	NS	Greater ↓ albuminuria + HR with cilnidipine
Kasliwal et al. (2016) [18]	Meta-analysis	Cilnidipine vs multiple	SMD ≈ -0.05	SMD ≈ -0.03	~0	NS	NS	↓ BP variability
AMCILAZ-EH (Present Study)	Prospective, multicentric real-world ABPM	Amlodipine vs Cilnidipine vs Azelnidipine	-19.1 ± 9.4 vs -15.2 ± 8.7 vs -13.1 ± 7.6	-10.3 ± 6.1 vs -7.9 ± 5.7 vs -7.1 ± 5.0	Aml vs Cil: -3.9; Aml vs Aze: -6.0; Cil vs Aze: -2.1	Overlapping CIs	0.287	No intergroup difference; trend toward greater awake SBP and DBP reduction with amlodipine compared to azelnidipine.

OBP reductions were generally greater than ABPM reductions across groups, reinforcing the known limitations of OBP measurements and the clinical value of ABPM [7,18]. Pulse rate remained largely unchanged with all three drugs, although azelnidipine demonstrated a numerical trend toward greater pulse-rate reduction. Similarly, nocturnal dipping showed a numerical improvement with azelnidipine, but this did not reach statistical significance, likely because of the limited sample size.

The study had several important limitations. A high attrition rate, largely related to limited tolerability and patient discomfort associated with serial ABPM, substantially reduced the effective sample size. Of the 113 patients screened, only 50 completed follow-up. This reduced the statistical power to detect small intergroup differences and increased the possibility of both false-positive and false-negative findings. Comparison between completers and excluded patients showed broadly similar baseline demographic and clinic BP characteristics, although excluded patients had modestly lower baseline ambulatory SBP values and higher pulse rate. Attrition-related selection bias therefore cannot be excluded. In addition, the observational, non-randomized design, physician-directed treatment allocation, non-standardized dose escalation, and absence of adjusted analyses introduce the possibility of residual confounding.

Overall, the findings of AMCILAZ-EH should be viewed as exploratory and hypothesis-generating. The study supports the observation that all three agents were associated with clinically meaningful short-term BP reduction in a real-world setting, but no definitive conclusions regarding comparative efficacy can be drawn. Larger randomized studies with standardized dosing, longer follow-up, and adequately powered ABPM-based assessment are needed to determine whether clinically meaningful differences exist among these agents in ambulatory and circadian BP control.

Limitations

The AMCILAZ-EH study has several important limitations. First, it was an observational, non-randomized study with physician-directed treatment allocation, introducing the possibility of selection bias and residual confounding. Second, the final sample size was modest (n = 50) and substantially lower than the originally planned cohort, limiting statistical power, particularly for detecting intergroup differences and changes in categorical outcomes such as dipping status. The unequal group distribution further increased the risk of type II error. A high attrition rate (55.7%), largely related to limited tolerability of ABPM and follow-up challenges, substantially reduced the effective sample size and represents an important limitation. Excluded patients demonstrated modestly lower baseline ambulatory SBP values and higher pulse rate compared with completers, suggesting the possibility of attrition-related selection bias.

Third, the follow-up duration was limited to eight weeks, which may have been insufficient to observe meaningful changes in circadian BP patterns, autonomic modulation, or long-term cardiovascular outcomes. In addition, dose titration was not standardized and was based on physician judgment and BP response, potentially introducing heterogeneity in treatment exposure. Dose-stratified or dose-adjusted analyses were not performed because of the limited sample size. Multiple comparisons were performed without formal adjustment, increasing the possibility of type I error; therefore, isolated statistically significant findings should be interpreted cautiously.

Although ABPM was performed using standardized devices, variability in patient adherence and daily activity patterns may also have influenced ambulatory measurements. Finally, the wide age distribution (18-80 years) may have introduced heterogeneity in vascular properties, since arterial stiffness and vascular compliance vary with age. Given that CCBs act predominantly through vasodilation, age-related differences in vascular elasticity may have influenced treatment response and ambulatory BP outcomes.

## Conclusions

In this multicentric real-world study, amlodipine, cilnidipine, and azelnidipine were each associated with significant reductions in clinic and ambulatory BP over an eight-week period. No consistent intergroup differences were observed across most BP parameters, although isolated differences in awake ambulatory BP were noted. These findings should be interpreted as exploratory, and no definitive conclusions regarding comparative efficacy or equivalence can be drawn given the observational design, modest sample size, and limited statistical power.

While azelnidipine showed a numerical improvement in nocturnal dipping pattern and a trend toward greater pulse rate reduction, cilnidipine showed a numerical trend toward better OBP control, but these observations were not statistically significant. Amlodipine demonstrated a numerically greater reduction in 24-hour SBP. Overall, the results suggest that short-term BP reduction may be broadly similar across agents; however, these findings require cautious interpretation and should not be considered evidence of equivalence. Larger, randomized studies with standardized dosing and longer follow-up are required to determine whether meaningful differences exist in circadian BP control, autonomic modulation, and long-term cardiovascular outcomes. The high dropout rates observed also highlight practical challenges with serial ABPM in real-world settings.
